# Efficacy of novel β_3_‐adrenoreceptor agonist vibegron on nocturia in patients with overactive bladder: A post‐hoc analysis of a randomized, double‐blind, placebo‐controlled phase 3 study

**DOI:** 10.1111/iju.13877

**Published:** 2018-12-17

**Authors:** Masaki Yoshida, Masayuki Takeda, Momokazu Gotoh, Osamu Yokoyama, Hidehiro Kakizaki, Satoru Takahashi, Naoya Masumori, Shinji Nagai, Keita Hashimoto, Kazuyoshi Minemura

**Affiliations:** ^1^ Department of Urology National Centre for Geriatrics and Gerontology Obu Aichi Japan; ^2^ Department of Urology Graduate School of Medical Sciences University of Yamanashi Kofu Yamanashi Japan; ^3^ Department of Urology Nagoya University Graduate School of Medicine Nagoya Aichi Japan; ^4^ Department of Urology Faculty of Medical Science University of Fukui Fukui Japan; ^5^ Department of Renal and Urologic Surgery Asahikawa Medical University Asahikawa Japan; ^6^ Department of Urology Nihon University School of Medicine Tokyo Japan; ^7^ Department of Urology School of Medicine Sapporo Medical University Sapporo Hokkaido Japan; ^8^ Kyorin Pharmaceutical Tokyo Japan

**Keywords:** β_3_‐adrenoreceptor agonist, hours of undisturbed sleep, nocturia, overactive bladder, vibegron

## Abstract

**Objectives:**

To investigate the efficacy of vibegron on nocturia in patients with overactive bladder.

**Methods:**

Among the Japanese overactive bladder patients enrolled in the placebo‐controlled, multicenter, randomized, double‐blind phase 3 study of vibegron, a total of 669 patients with nocturia (≥1 nocturnal void) were included. Changes from baseline in micturition parameters were compared for vibegron treatment (50 and 100 mg/day) versus placebo. Correlations of hours of undisturbed sleep with the frequency of nocturnal voiding and the volume of the first nocturnal voiding were examined. Demographics and baseline characteristics contributing to reduction in the frequency of nocturnal voiding were also analyzed.

**Results:**

At week 12, the frequency of nocturnal voiding was reduced from baseline by 0.74 and 0.78, respectively, for the vibegron 50 and 100 mg groups; the reductions were significant when compared with the placebo group (*P* < 0.05 and *P* < 0.001, respectively). The mean volume of nocturnal voids and the volume of the first nocturnal voiding were significantly greater in the vibegron groups than in the placebo group. The vibegron groups showed significant correlations of hours of undisturbed sleep with the changes in the frequency of nocturnal voiding and in the volume of the first nocturnal voiding. Vibegron treatment, no previous treatment with anticholinergics, ≥12 voids per day and hours of undisturbed sleep <180 min significantly contributed to a reduction in the frequency of nocturnal voiding.

**Conclusions:**

Vibegron is a useful therapeutic option for improving nocturia in patients with overactive bladder.

**Abbreviations & Acronyms**
BMI: body mass index
CI: confidence interval
FAS: full analysis set
HUS: hours of undisturbed sleep
LS mean: least squares mean
NPi: nocturnal polyuria index
OAB: overactive bladder
QOL: quality of life
REM: rapid eye movement
SE: standard error

## Introduction

OAB is a syndrome with urgency as a primary symptom, and is usually accompanied by urinary frequency, nocturia and sometimes urgency urinary incontinence.^1^ The prevalence of OAB in the general population is reported to range from 10% to 20% in Japan, Europe and the USA.^2^, ^3^, ^4^ Nocturia is defined as a complaint that a person affected has to awaken at least once at night to urinate.^1^ Among the lower urinary tract symptoms, nocturia remarkably disrupts the activities of daily living and QOL.^5^ Furthermore, nocturia has been reported to cause sleep interruption, and to be related to prevalence rates of depression symptoms and fracture as a result of fall.^6^, ^7^, ^8^ Nocturia has been reported to be caused by nocturnal polyuria, sleep disturbances, circadian clock disorders (e.g. circadian rhythm sleep disorder) and reduced bladder capacity.^9^, ^10^, ^11^ As OAB might attribute to reduced bladder capacity,^12^ in the treatment for nocturia associated with OAB, anticholinergics are used as a standard therapy.^13^, ^14^ However, anticholinergic therapy has several problems with a low adherence rate in continuing medication because of inadequate effectiveness and adverse drug reactions, such as dry mouth, constipation and cognitive impairment.^15^, ^16^, ^17^, ^18^ Therefore, alternatives to anticholinergic drugs are necessary.

β_3_‐Adrenoreceptors are predominantly expressed in human bladder tissue. A β_3_‐adrenoreceptor agonist causes smooth muscle relaxation in the bladder to increase the bladder capacity, and has attracted attention as a new therapeutic drug for OAB.^19^, ^20^, ^21^ Vibegron is a novel, potent and selective β_3_‐adrenoreceptor agonist, and exerts excellent pharmacological activity (*in vitro* and *in vivo*).^22^ Recently, a phase 3 study in Japanese OAB patients was carried out, and showed excellent efficacy and high tolerability of vibegron when compared with a placebo.^23^ In the present study, we carried out a post‐hoc analysis on nocturia using the data of patients in the phase 3 study, and evaluated the efficacy of vibegron on nocturia.

## Methods

### Patients

A phase 3 study of vibegron in Japanese patients with OAB (JapicCTI‐152936) was carried out as a placebo‐controlled, multicenter, randomized, double‐blind study, and its details were published.^23^ The eligibility criteria for the present study were as follows: those who visited the medical institutions participating in the study between July 2015 and June 2016; those aged ≥20 years who had suffered persistent OAB symptoms for >6 months at the time they gave their informed consent; and during a 2‐week placebo run‐in phase, OAB patients who met all of the following criteria: mean frequency of daily urgency episodes or urge urinary incontinence episodes ≥1.0, total frequency of urge urinary incontinence episodes exceeding half the total episodes of incontinence and the mean frequency of daily voiding ≥8.0. After the 2‐week run‐in phase, the eligible patients were randomly assigned, and were followed up for 12 weeks.

Among the OAB patients enrolled in the phase 3 study, those with one or more nocturnal voiding per night who were selected for vibegron treatment (at 50 mg/day or 100 mg/day) or placebo treatment were included in the present post‐hoc analysis.

### Outcome measures

For each micturition parameter (frequency of nocturnal voiding, volume per nocturnal void, volume of the first nocturnal voiding and HUS [defined as the length of time between when a patient goes to bed and when he/she first gets up at night to urinate]), changes from baseline (at week 0) to week 12 of treatment were compared between the vibegron and placebo groups, and the mean value calculated based on 3‐day frequency–volume chart before assessment was used in the analyses.

Correlations between changes from baseline to week 12 in the mean HUS and in the frequency of nocturnal voiding, as well as between changes from baseline to week 12 in the mean HUS and in the mean volume of the first nocturnal voiding were examined for the patients treated with vibegron active doses. In addition, demographic and baseline characteristics contributing to a reduction in the frequency of nocturnal voiding (decrease in the number of voids by ≥1) were identified.

### Statistical analysis

For the assessment of differences among groups in the baseline characteristics, the χ^2^‐test was used for categorical variables, and one‐way ANOVA was used for continuous variables.

For changes in individual micturition parameters from baseline to week 12, a constrained longitudinal data analysis model was used to calculate LS means and 95% CIs, and between‐group comparison was made.^24^ The statistical significance level was set at *P* < 0.05 for a two‐sided test. Multiplicity adjustment was not considered.

Spearman's rank correlation coefficients were calculated between changes in the mean HUS and in the frequency of nocturnal voiding, and between changes in the mean HUS and in the mean volume of the first nocturnal voiding.

For the purpose of identifying demographic and baseline characteristics contributing to a reduction in the frequency of nocturnal voiding, logistic regression models were used with the reduction from baseline to week 12 in the frequency of nocturnal voiding (≥1 episode) as a binary response variable in the following two steps. First, logistic regression analyses were carried out with the treatment groups of vibegron (integrated the two active doses) and placebo arm, and the individual demographic and baseline characteristics as explanatory variables. The factors affecting the reduction were extracted based on *P*‐values of the explanatory variables in the analyses. Second, multiple logistic regression analysis was carried out to estimate odds ratios of the reduction in the frequency of nocturnal voiding.

SAS software version 9.4 for Windows (SAS Institute, Cary, NC, USA) was used for the statistical analyses.

## Results

### Patient characteristics

Out of the 1232 patients enrolled in the phase 3 study and randomly assigned to one of the treatment arms, after excluding those for whom efficacy‐related data were missing or were considered irrelevant for any other reason, the FAS population consisted of 1224 patients. Among the FAS population, 669 patients were eligible for this post‐hoc analysis, in which 224 were assigned to the placebo group, 227 to the vibegron 50 mg group and 218 to the vibegron 100 mg group. Demographic and baseline characteristics of the micturition parameters of patients are shown in Table 1. Evaluation of all patients showed a greater proportion of women, mean age of approximately 60 years, hypertension noted in approximately 30% of patients and a NPi of <0.33 in approximately 60–70% of patients. The demographic and baseline characteristics were almost similar across the groups.

**Table 1:** Demographics and baseline clinical characteristics of patients with more than one nocturnal void per night

Treatment group	Placebo (*n* = 224)	Vibegron 50 mg (*n* = 227)	Vibegron 100 mg (*n* = 218)	*P*‐value
Women, *n* (%)	201 (89.7)	203 (89.4)	194 (89.0)	0.968
Age (years)	61.2 ± 11.4	59.9 ± 11.5	60.7 ± 11.0	0.477
BMI (kg/m^2^)	23.37 ± 4.15	23.15 ± 4.33	23.23 ± 3.98	0.847
Duration of OAB symptoms (months)	62.0 ± 63.6	56.8 ± 61.5	70.0 ± 79.1	0.122
No previous anticholinergic therapy for 1 year, *n* (%)	194 (86.6)	190 (83.7)	195 (89.4)	0.206
Hypertension vital sign†, *n* (%)	69 (30.8)	68 (30.0)	66 (30.3)	0.981
Nocturnal polyuria (NPi ≥0.33), *n* (%)	81 (36.2)	71 (31.3)	85 (39.0)	0.226
Voided volume/24 h (mL)	1746.69 ± 434.54	1720.65 ± 439.27	1714.66 ± 412.80	0.705
NPi	0.316 ± 0.098	0.308 ± 0.090	0.315 ± 0.095	0.577
No. voids/24 h	11.74 ± 2.44	11.74 ± 2.40	11.54 ± 2.42	0.608
No. nocturnal voiding/24 h	1.75 ± 0.80	1.69 ± 0.77	1.69 ± 0.78	0.666
Volume per nocturnal void (mL)	204.3 ± 69.5	202.4 ± 72.1	206.8 ± 75.8	0.813
Volume of the first nocturnal voiding (mL)	208.04 ± 95.51	210.52 ± 102.65	201.06 ± 94.08	0.570
HUS (min)	182.6 ± 74.6	185.8 ± 75.4	172.0 ± 69.7	0.117

Data represent the mean ± standard deviation. †SBP ≥140 mmHg or DBP ≥90 mmHg.

### Changes from baseline in micturition parameters

**Figure 1:**
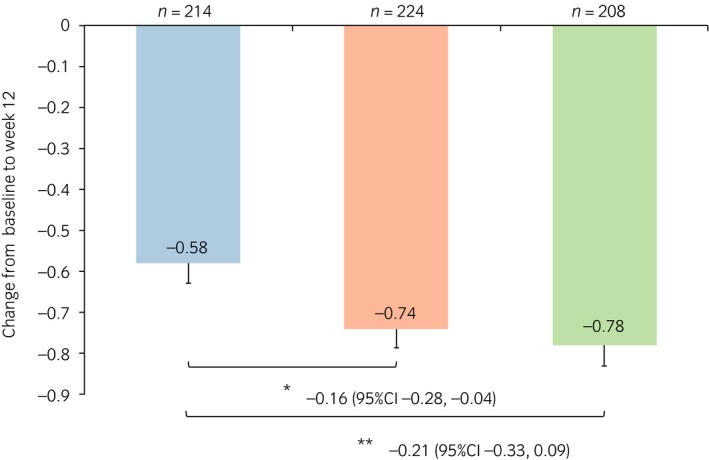
Change in frequency of nocturnal voiding per 24 h. Blue, red and green bars show the placebo, vibegron 50 mg and vibegron 100 mg groups, respectively. *P < 0.05; **P < 0.001.

**Figure 2:**
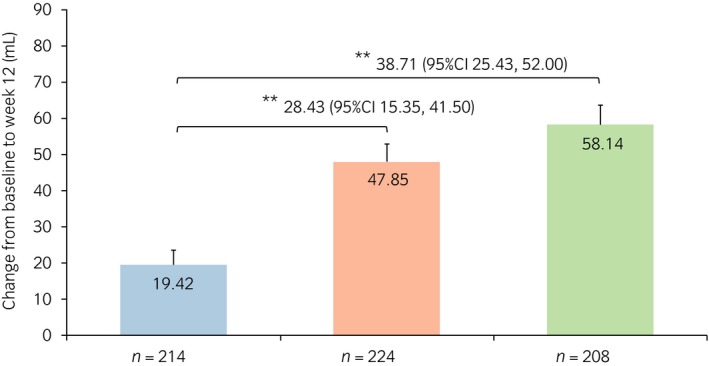
Change in volume per nocturnal void. Blue, red and green bars show the placebo, vibegron 50 mg and vibegron 100 mg groups, respectively. **P < 0.001.

**Figure 3:**
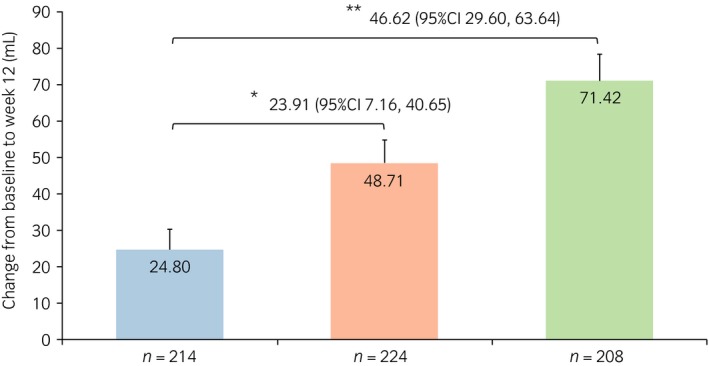
Change in volume of the first nocturnal voiding. Blue, red and green bars show the placebo, vibegron 50 mg and vibegron 100 mg groups, respectively. *P < 0.05; **P < 0.001.

**Figure 4:**
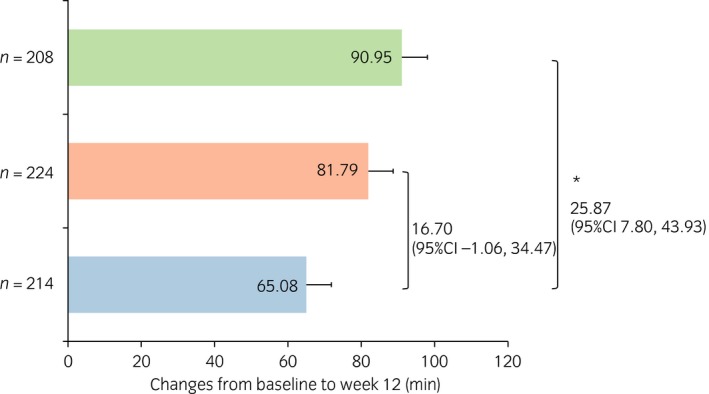
Change in HUS. Blue, red and green bars show placebo, vibegron 50 mg and vibegron 100 mg groups, respectively. *P < 0.05.

The change from baseline to week 12 in the mean frequency of nocturnal voiding was −0.74 and −0.78 for the vibegron 50 and 100 mg groups, respectively, both of which were significantly greater than that of the placebo group, −0.58 (*P* = 0.007 and *P* < 0.001, respectively; Fig. 1). The change from baseline in the mean volume per nocturnal void was 47.85 and 58.14 mL for the vibegron 50 and 100 mg groups, respectively, both of which were significantly greater than that of the placebo group, 19.42 mL (*P* < 0.001 for both; Fig. 2). The change from baseline in the mean volume of the first nocturnal voiding was 48.71 and 71.42 mL for the vibegron 50 and 100 mg groups, respectively, both of which were significantly greater than that of the placebo group, 24.80 mL (*P* = 0.005 and *P* < 0.001, respectively; Fig. 3). The change from baseline in the mean HUS was longer in the vibegron 50 mg group with 81.79 min than in the placebo group with 65.08 min, although no significant difference was noted between the two groups (*P =* 0.065). In contrast, a significant extension of HUS in 90.95 min was observed in the vibegron 100 mg group compared with the placebo group (*P* = 0.005; Fig. 4).

### Correlations of changes in HUS with changes in the frequency of nocturnal voiding and in the volume of the first nocturnal voiding

**Figure 5:**
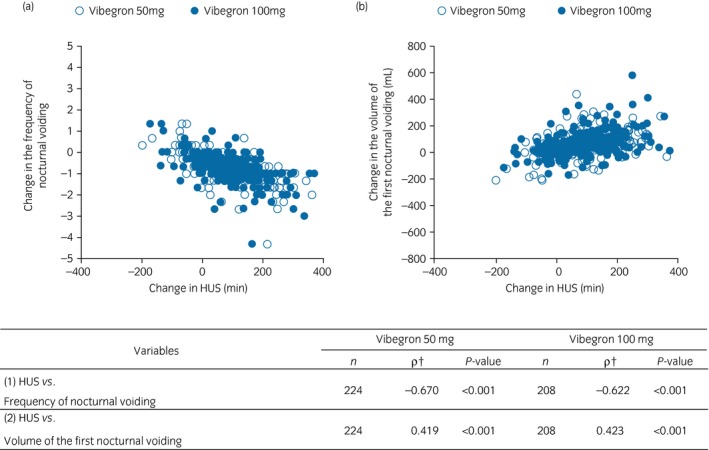
Correlations of changes in HUS with change in the frequency of nocturnal voiding and in the volume of the first nocturnal voiding. †Spearman's rank correlation coefficient.

Figure 5 shows negative correlations between changes from baseline to week 12 in the mean HUS and the frequency of nocturnal voiding (vibegron 50 mg group: ρ = −0.670, *P* < 0.001; vibegron 100 mg group: ρ = −0.622, *P* < 0.001), and positive correlations between change from baseline to week 12 in the mean HUS and in the mean volume of the first nocturnal voiding (vibegron 50 mg group: ρ = 0.419, *P* < 0.001; vibegron 100 mg group: ρ = 0.423, *P* < 0.001).

### Demographic and baseline characteristics contributing to a reduction in the frequency of nocturnal voiding

**Figure 6:**
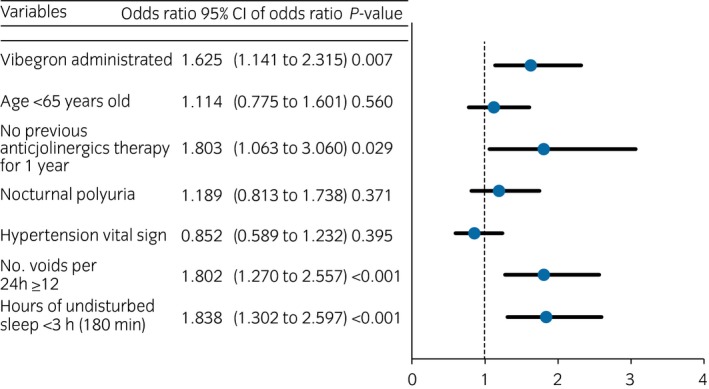
Odds ratios of demographic and baseline characteristics associated with a reduction in the frequency of nocturnal voiding. Circles show odds ratio and the bars show 95% CIs.

The multivariate analysis showed that the following variables significantly contributed to a reduction in the frequency of nocturnal voiding, in addition to vibegron treatment: no previous treatment with anticholinergics for the past 1 year; ≥12 voids per day; and HUS <3 h (180 min; Fig. 6).

## Discussion

Vibegron is a potent, highly selective β_3_‐adrenoreceptor agonist. The phase 3 study of OAB patients that was carried out in Japan has shown that vibegron is effective in improving OAB symptoms and is highly tolerable.^23^ In the present post‐hoc analysis using the data derived from the phase 3 study, the efficacy of vibegron in managing nocturnal frequency in OAB patients was evaluated. This analysis evaluated the efficacy of vibegron (a new β_3_‐adrenoreceptor agonist) on nocturia and related parameters in patients with OAB.

The reduced frequency of nocturnal voiding by vibegron treatment might be explained by the increased bladder capacity as a result of smooth muscle relaxation, which is caused through activation of β_3_‐adrenoreceptors expressed in the bladder tissue. The effects of anticholinergics, which inhibit contractions of the bladder smooth muscle, in improving nocturia in Japanese OAB patients were also reported.^14^, ^23^ The changes from baseline in the frequency of nocturnal voiding, the volume per nocturnal void and HUS in the vibegron treatment arm, which were assessed by comparison with the placebo treatment arm, determined in the present post‐hoc analysis were almost similar to those reported in previous studies of anticholinergic agents.^14^, ^23^

Chappel *et al*. reported in a phase 2 trial that mirabegron 50 mg significantly reduced nocturia episodes by −0.22 versus placebo.^25^ Mirabegron 100 mg reduced nocturia episodes by −0.04 versus placebo, but the difference was not significant. Yamaguchi *et al*. also reported in a phase 3 trial that 12‐week treatment with mirabegron 50 mg reduced nocturia episodes by −0.44, but the difference was not significant versus placebo.^26^ In the present study, a significant decrease was observed both in the vibegron 50 mg group (−0.74) and in the 100 mg group (−0.78) versus the placebo group. As for safety profile, those of vibegron and mirabegron are almost identical based on the phase 3 study of each drug.

In addition, rates of previously treated patients were 42.6% in the mirabegron phase 2 trial^25^ and 63.1% in the phase 3 trial,^26^ whereas the rates were as low as 16.3% in the vibegron 50 mg group and 10.6% in the 100 mg group in the present study.

Although no head‐to‐head comparison data are available between vibegron and mirabegron, the above data suggest that the presence/absence of pretreatment might have influenced the effects of vibegron and mirabegron on the frequency of nocturnal voiding.

Mirabegron, a CYP2D6 inhibitor, has been reported to have off‐target effects,^27^ whereas vibegron is metabolized independently from CYP3A4, 2D6 or 2C9, and is thus considered less likely to cause drug–drug interactions. Vibegron is also reported to be highly selective to cardiac ion channels (hERG, hCav1.2 and hNav1.5) and serotonin transporters.^22^

The change from baseline to week 12 in HUS was extended by 81.8 min for the vibegron 50 mg group and by 91.0 min for the vibegron 100 mg group (*P* = 0.065 and *P* = 0.005, respectively, compared with the placebo group). Significant correlations were noted between the extended HUS and the reduced frequency of nocturnal voiding, as well as the increased mean volume of the first nocturnal voiding. It is therefore considered that the reduced frequency of nocturnal voiding and the increased volume per nocturnal void, especially an increase in volume of the first nocturnal voiding, led to the extension of HUS.

Nocturia has a great impact on sleep, and there are several reports showing that nocturia impairs the patient's QOL.^2^ Non‐REM episodes are reported to be concentrated in the first 3 h of sleep.^28^ The time when the patient gets up for the first time to urinate; that is HUS, is important to ensure the quality of sleep.^29^ It has also been suggested that the first 3 h of sleep contribute to the quality of the sleep.^28^ The HUS in the vibegron 100 mg group was 2.9 h (172.0 min) before treatment and 4.4 h (267.0 min) at week 12, showing that HUS was extended to the range of time required to ensure the quality of sleep.^29^ The HUS in the vibegron 50 mg group was 3.1 h (185.8 min) before treatment and 4.4 h (264.6 min) after treatment, indicating that vibegron extended HUS to longer than 4 h. The extension in this group was not statistically significant, but was of great clinical meaning. The present results suggest a possibility that vibegron might reduce the frequency of nocturnal voiding, resulting in improvement of the quality of sleep.

The multivariate analysis on demographic and baseline characteristics contributing to a reduction in the frequency of nocturnal voiding revealed the following variables: vibegron treatment; the absence of previous treatment with anticholinergics for the past 1 year; ≥12 voids per day; and HUS <3 h (180 min). However, age, nocturnal polyuria and hypertension had no impact on the reduction in the frequency of nocturnal voiding. On the basis of these results, vibegron therapy by itself is expected to improve nocturia, and vibegron might be more beneficial for patients who have not received anticholinergic therapy for the past 1 year, those who suffer from severe urinary frequency and those who have to get up to urinate soon after falling asleep.

There are several limitations of this post‐hoc analysis, because it was an exploratory analysis of data obtained in the phase 3 study. Patients had a lower frequency of nocturnal voiding because the primary end‐point was not nocturia in the phase 3 study. Furthermore, QOL evaluation on nocturia was not adequately carried out. In future studies, QOL needs to be evaluated using N‐QOL or other questionnaires specific to nocturia, and investigation using the Pittsburgh Sleep Quality Index or other relevant questionnaires is necessary to evaluate the relationship with the quality of sleep.

In conclusion, the present post‐hoc analysis further revealed that vibegron reduced the frequency of nocturnal voiding, and increased the volume per nocturnal void and the volume of the first nocturnal voiding in OAB patients with one or more nocturnal void. In addition, vibegron might improve the quality of sleep as a result of the extension of HUS. Vibegron can be a new treatment option for improving nocturia in OAB patients.

## Conflict of interest

Masaki Yoshida has received consultancy fees from Kyorin; grants from Astellas; and speaker fees from Kyorin, Kissei, Astellas, Daiichi‐Sankyo, Ono and Pfizer. Masayuki Takeda has received consultancy fees from Kyorin; grants from Astellas, Asahi‐Kasei Pharma, GSK, Nippon Shinyaku, Takeda and Pfizer; and speaker fees from Kyorin, Kissei, Astellas, Daiichi‐Sankyo, Nippon Shinyaku, Ono and Pfizer. Momokazu Gotoh has received consultancy fees from Kyorin, Medtronics Japan and Taiho; grants from Kyorin, Kissei, Asahi‐Kasei Pharma, Astellas, Chugai, Daiichi‐Sankyo, Nippon Shinyaku, Novartis, Ono, Pfizer, Sanofi‐Aventis, Taiho and Takeda; and speaker fees from Kyorin, Kissei, Asahi‐Kasei Pharma, Astellas, AstraZeneca, Daiichi‐Sankyo, Hisamitsu, Nippon Shinyaku, Ono, Pfizer, Sanofi‐Aventis and Takeda. Osamu Yokoyama has received consultancy fees from Kyorin, Astellas, GSK, Pfizer and Taiho; grants from Kissei, Astellas, Nippon Shinyaku, Ono, Pfizer and Taiho; and speaker fees from Kissei, Astellas, Nippon Shinyaku and Pfizer. Hidehiro Kakizaki has received consultancy fees from Kyorin, Astellas and Taiho; grants from Kissei, Astellas, Daiichi‐Sankyo, Nippon Shinyaku, Taiho and Takeda; and speaker fees from Kyorin, Kissei, Astellas, Nippon Shinyaku and Pfizer. Satoru Takahashi has received consultancy fees from Kyorin; grants from Astellas and Nippon Shinyaku; and speaker fees from Kyorin, Kissei, Astellas, Nippon Shinyaku and Pfizer. Naoya Masumori has received consultancy fees from Kyorin; research grants from Astellas, Daiichi‐Sankyo, MSD, Ono and Taiho; and received lecture fees from Kyorin, Kissei, Janssen, Nippon Shinyaku, Takeda and Vorpal Technologies. Shinji Nagai, Keita Hashimoto and Kazuyoshi Minemura are employees of Kyorin.
